# Asiatic Cholera, an Extract from a Paper Read before the Chicago Medical Society

**Published:** 1852-11

**Authors:** E. McArthur


ARTICLE III.—*Asiatic Cholera, an extract from a paper read to the Chicago Medical Society, by* E. McArthur, M. D.

We have now related in detail, 34 cases, in 25 different families. In nine families, we have noticed the second attack; in five, we have found the third; and in one, the fourth. We have been unable to trace the disease from either of the 25 families, to any others, with the exception of two. In 24 of the 25 families, we have been unable to trace it to any exposure to cholera patients whatsoever.

In the full conviction that Asiatic Cholera partakes more of an epidemic than of a contagious character, I have selected these cases as evidences, to substantiate, as far as they may, in the minds of others, the truthfulness of these convictions. That there have been cases in this community of Asiatic Cholera which, viewed without a due regard to surrounding influences, and circumstances, might, upon a superficial view, *seem* to show a contagious character, I will not for a moment deny. But when it is remembered that *the whole of this community felt the enervating influence of a latent agent*, *affecting the entire physical and mental man*, it would seem almost superfluous to assign as a direct cause of attack, a contagious influence. It is well known to the medical profession in general, in this part of the country, that this *latent influence was universal* during the time that cholera prevailed, and if an individual could be found here and there, who did not feel its influence, this would but serve to show the correctness of the general proposition.

This may not be so well understood where this fell disease has not prevailed. But in this community, or any other where cholera has extensively prevailed, it is but too well understood.

Some suppose that because many cases of this disease have ultimately become developed after having been exposed to patients having it, that *therefore* it is contagious. We might with the same propriety suppose that in malarious districts, when intermittent fevers prevail, and one patient after another is prostrated by it, after having been exposed to patients sick with fever, that consequently intermittent fever is contagious.

But who supposes that the whole class of malarious diseases is contagious. ?

And yet it is well known that where individuals living in these miasmatic districts expose themselves either to the sudden changes of atmospheric influences, or by excessive labor and watchings debilitate themselves, they are more likely to suffer from attacks of fever than those who are cautious, temperate, and careful in their daily living and deportment.

And it is equally *as well* known that individuals living in districts where cholera prevails, who are calm, quiet, judicious in their living, temperate, and in good health, it matters not whether they are nursing and taking care of cholera patients, or attending to ordinary occupations; they generally escape the disease. On the other hand, those who are excited, intemperate, injudicious in their living and habits, debilitated and alarmed, are very likely to be taken with it, and especially if they come in contact with the disease.

And here the question very naturally arises, whether it is communicated to them by the principle of contagion, or whether the increased debility, and intemperance, the anxiety and alarm, does not serve as an *exciting cause* to develop that which is already *within them*, in a latent state.

Thus it does appear that the epidemic influence operates upon the community in a *secret* and *invisible* manner, as a predisposing cause, and only wants some or all of the causes above detailed, to *develop* and bring it into action.

If this important question can be settled in the minds of the profession, and, through the profession, the common people become instructed in relation to the weight of the testimony bearing upon this point, a very great influence will, in this manner, be brought to bear upon the public mind, and as a consequence, this scourge of humanity will be divested of a large share of its terror. Who does not know the consequences resulting from the alarm and agitation that has resulted in many places where cholera has appeared?

The public mind has been a *perfect foment* in some places, and the consequence has been that the latent influence has been in *this way* developed to a fearful and alarming extent.

But how many have fallen victims to this man destroyer, who have never been exposed in any other way, than by the portion of atmospheric air inhaled into the lungs, and the terror and alarm that have continually preyed upon their minds?

Let the impression be generally entertained that this disease is strictly epidemic, or principally so, and the consequences will be that the sick will have better attention paid to them, and the living will have less alarm, and at the same time the bills of mortality will be greatly diminished.

We will make a few extracts from authors and writers of acknowledged celebrity and distinguished attainments, and close this already too lengthy article. Watson's Practice, page 808, in an enclosed paragraph. Dr. Condie remarks: "During the prevalence of the cholera in Philadelphia, in 1832, we closely investigated every fact calculated to throw light upon the question of its contagious or non-contagious character; and for this investigation our position in the Board of Health, and as chief in a large hospital, afforded us ample opportunities; but we were unable to discover the slightest evidence of the disease having been, in any one instance, communicated from the sick to the well."

Wood's Practice, volume I, page 654. "The hypothesis that cholera is propagated by contagion, has not been without numerous advocates. In favor of this, the facts have been adduced, 1. That it traveled along the lines of commercial intercourse, as in the course of large streams, in the tracks of caravans, and from port to port, across seas or oceans. 2. That its rate of advance never exceeded the possible rapidity of human progression. 3. That its occurrence often succeeded the arrival of an infected ship, caravan, or corps of marching soldiers. 4. That its attack was not general in the beginning, but, whether a city or particular family, one or a few were seized at first, and then others successively, as if the cause had passed from individual to individual. But it will be perceived that these were not proofs of contagion, but only evidences, so far as they went, of the possibility, or at best, of the probability of its existence; and it is not difficult to explain them without recourse to such a hypothesis. Thus, if the disease preferred the course of streams, it might have been in consequence of the known affinity of its cause for low, damp, and marshy situations; if it was observed especially along the routes of inland trade, this might have been owing to the circumstance that the largest and most conspicuous towns, which it was apt to select, are upon these routes, while remote spots which it also visited, are less open to observation; if it passed from an infected port to other transmarine ports, between which there was commercial intercourse, the fact could scarcely have been otherwise if it traveled at all, so intimately are all parts of the world associated in the meshes of a universal traffic; if its arrival was often preceded by that of a ship, or that of travelers from an infected place, this might well have been the result of accident, and very often no such connection could be traced, while intercourse of the kind was constantly carried on, without the consequence of the disease; and finally, if it sometimes attacked one person after another, instead of all simultaneously, this is nothing more than often occurs in the operation of morbid causes, confessedly non-contagious, and was not always true of cholera, which occasionally prostrated multitudes at one blow."

Eberle's Practice, volume II, page 55. "Where the evidence of contagion is so slight, therefore, that the most careful and judicious are led to entertain strong doubts of its existence, it is manifestly the duty of those whose station gives them an influence over public opinion, to discourage the belief in the prevalence of contagion. If the authority of those who have witnessed epidemic cholera, is to be taken as an evidence on this point, the foundation for the opinion of its contagious character, is but very slight. It is stated that in India, ninety-nine out of one hundred physicians, believe that cholera is *not* contagious; and in every country and district that has been invaded by this disease, a great majority of the most experienced and enlightened of the profession, entertain the same conviction."

Professor C. B. Coventry, on epidemic cholera, pages 38 and 39. "When we remember the panic which prevailed in 1832, the vexation caused, and the money expended in useless quarantines; the hurried and barbarous interment of the dead, scarce waiting till the breath had left the body, the worse than brutal desertion and neglect of friends and relations, all growing out of a belief in the contagious nature of the disease; we rejoiced in the belief that whatever of suffering a superintending Providence might see fit to inflict, we should not again thus aggravate them by our own acts.

If there was any one fact connected with the disease, which was immutably settled, as we supposed, it was its non-contagious character. If the decisive and explicit testimony of Annesly, who, for five years, had charge of a hospital where cholera patients were mingled indiscriminately with those from other diseases, and who tells us that not more than six or seven cases originated in the hospital during that time; the no less emphatic testimony of Dr. Searl, who had the disease in India, and who informed us that when in charge of the hospital at Warsaw, but two cases originated in the hospital, and that he himself slept in the bed in which a gentleman had died of the disease, the preceding night, without his contracting it.

If the testimony of Annesly and others, in India, that "when the disease attacked a corps in the army, it was the soonest got rid of by separating them into small detachments, * * * * * we repeat, if these facts, together with the experience of the profession from Calcutta to Moscow, and from Moscow to Quebec, and from Quebec to New Orleans, is not sufficient to settle the question of contagion, we are at a loss to see how it is ever to be settled."

Page 40. "The Health Commissioners in England are decidedly opposed to the doctrine of its promulgation by contagion." The Committee of the Royal College of Physicians, London, say: "Cholera appears to have been very rarely communicated by personal contact, and all attempts to stay its progress by cordons or quarantines, have failed." * * * *

Dr. Searl says: "Upon the question whether cholera is infectious or not, I can speak decidedly that it is not so! This is no vague opinion, hastily arrived at, but the deliberate result of grave consideration, and lengthened observation. * * *

I shall briefly adduce a few facts corroborative of the opinion expressed. First observing that, when in Poland, the principal cholera hospital of Warsaw, of which I was in charge, was on the skirts of the city, and the rendezvous of all the incurables within it, the professionals of the city sending me all their hopeless cases, and I had from thirty to sixty cases constantly under treatment, of which number half a dozen or more were buried daily.

"Well, then, of thirty or more attendants, during the three months that I was in charge, we had among this number only two cases of the disease; and the cause of the attack in both cases was most satisfactorily to be explained." And again, on page 43 and onward, Dr. Searl continues: "There is another fact, which alone, in my opinion, is sufficient to decide the question, namely; that contagious diseases are, one and all of them, characterized by the fact that the poison, or infectious matter of the disease, is concocted or developed in the system under circumstances of quickened circulation of the blood and depraved secretions—under the conditions, in short, of fever; whereas the condition of the system in cholera, and characteristic features of the disease, are precisely of the opposite character—inertia, general prostration, cold, defective excitement."

Dr. Milroy, as quoted by Professor Coventry, says, page 47: "As surely has cholera always sought out, and settled down upon the abodes of misery and filth, in every city of Europe, that has been visited by it, as the vulture-crows in the East, ever congregate where there is the most offal and garbage to be found."

Professor Spencer, on Asiatic Cholera, page 77, says: "If we regard the universal diarrhœa as a part of the epidemic, this should be equally contagious as at the more advanced periods.—Now, the prevalent diarrhœa simultaneously attacked the inhabitants at immense distances from each other, and almost the entire population of whole towns and villages were affected with this mild form of the disease.

Contagious complaints generally attack in small scouting parties, while epidemics, like immense armies, spread simultaneously over immense tracts of country.

Again, on page 78: "Contagious diseases rarely recur, while this epidemic would often return on the slightest application of the ordinary exciting causes. This disease could generally be cut short when attacked with appropriate remedies; entirely differing, in this respect, from contagious complaints, which proceed under any treatment. Contagious diseases do not require the influence of exciting causes, to develop them. The severe form of this disease rarely, if ever, attacked any one without some obvious exciting cause.

Professor Spencer says, page 80: "It may likewise be remarked, that those who went among the diseased were no more subject to the attacks than those who entirely avoided exposure. Indeed, several cases occurred under my own observation, among those who sedulously avoided their sick neighbors; becoming the ready victims of the disease, while not a single instance of death occurred among those who fearlessly and faithfully performed their duty.—Watchers lay down, and even slept on the same bed with the sick, without contracting the disease. * * * Physicians and nurses have been no more subject to attack than others. * * * * During, for instance, the English campaigns in India, two regiments being for a time stationed at different places, would be brought together, and daily and unceasingly intermingle, and yet the soldiers of one regiment would almost all have the disease, while those of the other would almost escape." *

(*To be continued.*)

---

* Bombay Report.

